# A safety type of genetically engineered bacterium that degrades chemical pesticides

**DOI:** 10.1186/s13568-020-00967-y

**Published:** 2020-02-18

**Authors:** Qin Li, Jing Li, Ke-Lai Kang, Yi-Jun Wu

**Affiliations:** 1 Laboratory of Molecular Toxicology, State Key Laboratory for Integrated Management of Pest Insects and Rodents, Institute of Zoology, Chinese Academy of Sciences, 1-5 Beichenxilu Road, Beijing, 100101 People’s Republic of China; 2 Environmental Standards Institute, Chinese Research Academy of Environmental Sciences, Beijing, 100012 People’s Republic of China

**Keywords:** Genetically engineered bacteria, Pesticide, Degradation, Carboxylesterase B1, Green fluorescent protein, Suicide

## Abstract

Chemical pesticides are used widely and their residues are found in the environment. Pesticide pollution has become a global problem. To find an economical, effective and safety way to degrade residues of pesticides in environment, we constructed a genetically engineered bacterium (GEB) having the ability to degrade pesticides, emit green fluorescence and has a containment system by using a dual plasmid expression system. One plasmid contains the genes of enhanced green fluorescent protein (EGFP) and carboxylesterase B1 (CarE B1), which were cloned downstream of lambda P_L_ promoter and expressed constitutively. The gene of CarE B1 encodes an insect-detoxifying enzyme possessing the degradability to organochloride pesticides, organophosphorus pesticides, carbamates, and pyrethoid insecticides. The other is the conditional suicide plasmid for containment system, in which the lethal gene used was the nuclease gene of *Serratia marcescens* without the leader-coding sequence and was placed downstream of T7 promoter. The GEB has wide prospects of application on cleanup of pesticide residues with its degradability to several pesticides and containment system.

## Introduction

Chemical pesticides are used widely in agriculture and forestry to control pests and diseases. Globally 4.6 million tons of chemical pesticides are annually sprayed into the environment, while only 1% of the sprayed pesticides are effective, and 99% of the pesticides applied are released to non-target soils, water bodies, and atmosphere, and finally absorbed by almost every organism (Zhang et al. 2011). Besides the sprayed pesticides, large amounts of unused and obsolete pesticides are threatening the environment and public health in many countries too (Karstensen et al. 2006). Pesticides residues have been found in various environment media and livings, and pollution of which has become a persistent environmental problem internationally (Reimer and Prokopy 2012; Akoto et al. 2013; Yurtkuran and Saygı 2013). The values for the permissible limits of some pesticides have been reported; for example, the reference doses (RfD) of pesticides fenpropathrin and permethrin are 0.025 and 0.05 mg/kg body weight/day, respectively (USEPA 1987, 1994); the acceptable daily intake (ADI) of pesticide chlorpyrifos is 0.01 mg/kg body weight/day (USATSDR 1997). Therefore, it is necessary to study the removal or degradation methods of pesticide residues in the environment. Among various technologies studied to clean up residual pesticides, such as chemical oxidation, biotreatment, catalytic treatment, and filtration (Wu et al. 2007), biodegradation was considered an environment friendly approach. Many genetically engineered microorganisms (GEMs) capable of biodegrading pesticides were constructed by recombinant DNA technology (Ingham et al. 1995; Lan et al. 2006; Chungjatupornchai et al. 2011; Khodi et al. 2012). However, the application of the GEMs is usually limited by the risk of genes moving from GEMs to other organisms and their potential impact on the distribution and growth of the indigenous microbial population. Therefore, it is needed to set up a system to track down the GEMs released into the fields and control the survival of GEMs.

Over the past few decades, several methods such as gene probes, PCR, monoclonal antibodies, bioluminescence, and autofluorescent proteins (AFPs), have been developed to monitor the GEMs (Matheson et al. 1997; Khan et al. 1998; Ramos-González et al. 1992; Ripp et al. 2000; Gory et al. 2001). Of these, AFPs demonstrates the unique superiority; it does not need additional substrates, cofactors, or proteins but O_2_ for fluorescence (Larrainzar et al. 2005). Green fluorescent protein (GFP) is a natural protein expressed in bioluminescent jellyfish that can emit bright green fluorescence after UV light excitation (Shimomura et al. 1962). Enhanced green fluorescent protein (EGFP) is a red-shifted variant of GFP, which has much stronger fluorescence and was often used in studies (Cormack et al. 1996). To control the survival of GEMs, some active biological containment systems have been designed, often based on lethal genes triggered by specific physical or chemical signals (Knudsen and Karlström 1991; Jensen et al. 1993; Szafranski et al. 1997; Balan and Schenberg 2005), but few of these systems were applied to GEMs whether in experiment or in field.

In this study, we described the construction of a genetically engineered bacterium (GEB) with the ability to degrade pesticides, emit green fluorescence and control itself survival by the co-transformation of two plasmids containing different functional genes and promoters, which provides a safe and effective method of pesticide biodegradation. Among the two plasmids used in this GEB, one was a plasmid carrying the fusion genes of EGFP and carboxylesterase B1 (CarE B1) of *Culex pipiens quinquefasciatus*, which were placed downstream of lambda P_L_ promoters and expressed freely without needing for inducer; the other was the compatible conditional suicide plasmid pDS containing two copies of the lethal nuclease gene of *Serratia marcescens* without the leader-coding sequence (Li and Wu 2009). The CarE B1 gene encodes an enzyme that confers pesticide resistance (Mouches et al. 1990). Studies indicated that CarE B1 has a strong degradation ability to various pesticides, such as organochloride pesticides, carbamates, pyrethoid insecticides, and organophosphorus pesticides (Vontas et al. 2000; Barata et al. 2004; Nishi et al. 2006).

## Materials and methods

### GEB construction

The *egfp* gene without stop codon amplified from pEGFP-N3 (Clontech, Palo Alto, CA, USA) by PCR was cloned into the plasmid pBV220 (kindly gifted by Ms R. Liu, Tianjin Medical College, China) (Primers used were No. 1 and No. 2 in Table 1) between *Eco*RI and *Bam*HI to generate plasmid pBV-EGFP. The PCR-amplified fragment of the whole sequence of pBV-EGFP with deletion of the repressor protein *cIts857* gene that is responsible for transcriptional regulation on plasmid pBV220 (Primers used are No. 3 and No. 4 in Table 1) was self-circularized with T4 DNA ligase to form the plasmid pL-EGFP.

**Table 1:** Primers used in this study

No	Restriction enzymes^a^	Sequence (5′ → 3′)^b^
1	*Eco*RI	GGGAATTCATGGTGAGCAAGGGCGAGGAGCT
2	*Bam*HI	TTGGATCCCTTGTACAGCTCGTCCATGCCGAG
3	*Sac*I	TTCGAGCTCCGTGCGTGTTGACTATTTTACCT
4	*Xba*I	GCTCTAGATCGGCAAGGTGTTCTGGTC
5	*Bgl*II	GAAGATCTATGAGTTTGGAAAGCTTAACCGT
6	*Pst*I	AACTGCAGTCAAAACAGCTCATCATTCACGTACAT

^a^Restriction enzyme that can digest the sequences underlined

^b^Underlined sequences are restriction sites

**Fig. 1:**
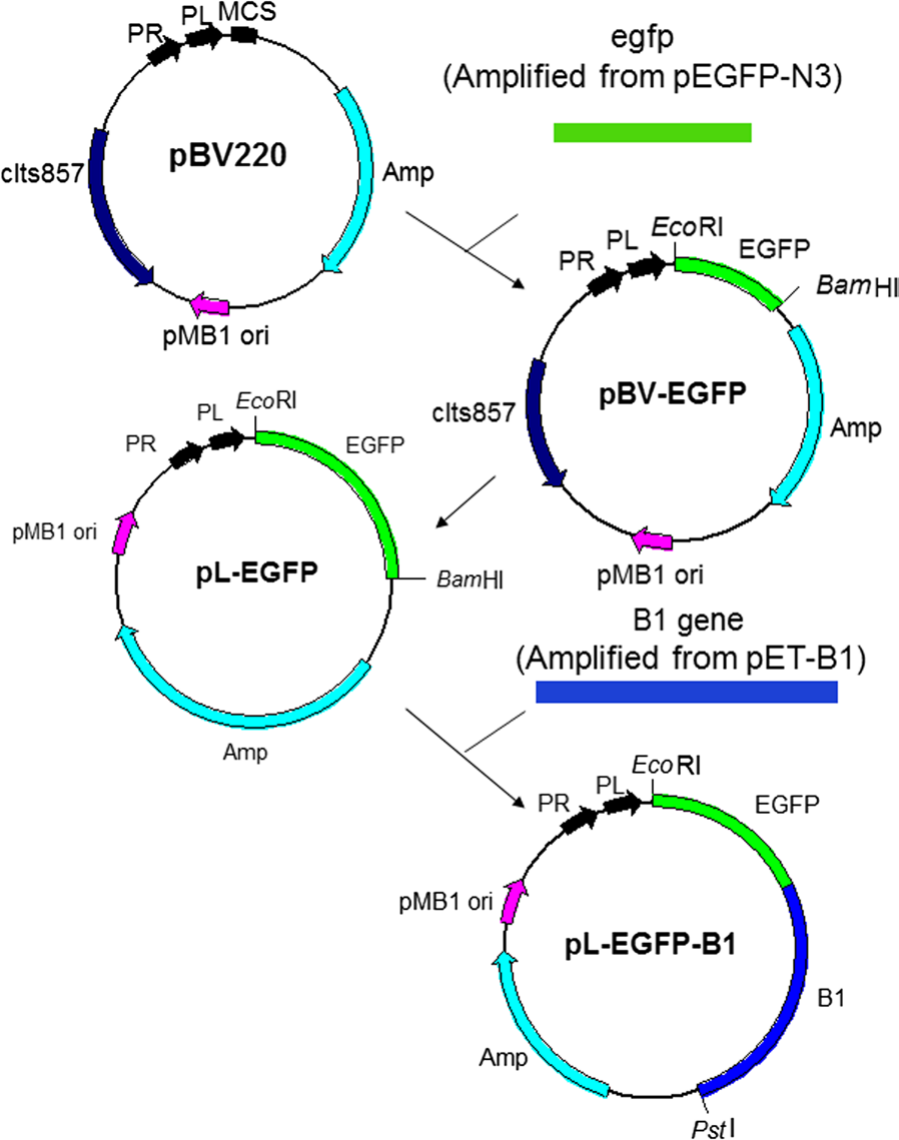
Construction map of the recombinant plasmid. Construction map of the plasmid pL-EGFP-B1. The *egfp* gene without the stop codon was cloned into the plasmid pBV220 to construct the plasmid pBV-EGFP. The entire sequence of pBV-EGFP except the *cIts857* gene was amplified by PCR and self-circularized to generate the plasmid pL-EGFP. Then, the B1 gene was inserted to generate the final plasmid pL-EGFP-B1. P_R_ and P_L_ indicate the lambda P_R_ promoter and P_L_ promoter, respectively. MCS is the multiple cloning sites and ori is the origin

The carboxylesterase B1 gene was amplified by using primers No. 5 and No. 6 (see Table 1) from pET-B1, which was obtained from Dr. C. L. Qiao (Institute of Zoology, Chinese Academy of Sciences, Beijing, China). The PCR-amplified B1 gene was then inserted between the *Bam*HI and *Pst*I sites of pL-EGFP to obtain the plasmid pL-EGFP-B1 (Fig. 1b).

The GEB (hereafter, BL21AI-GBS) that can emit green fluorescence, degrade pesticides and commit suicide when induced was constructed by transforming the above plasmid pL-EGFP-B1 and another plasmid pDS sequentially into *E. coli* strain BL21-AI^TM^ (*F*^−^*ompT hsdS*_*B*_*(r*_*B*_^−^*m*_*B*_^−^*) gal dcm araB::T7RNAP*-*tetA*, Invitrogen) through the calcium chloride procedure (Sambrook and Russell 2001). pDS was a conditional suicide plasmid induced by arabinose, containing two suicide cassettes designed with the nuclease gene of *Serratia marcescens* (Li and Wu 2009). BL21AI-GBS’s green fluorescence and ability to degrade pesticides do not require induction, but the cell suicide containment system must be activated by adding arabinose to the culture medium.

### Detection of the intensity of fluorescence and GEB growth curve

Cultures incubated overnight were transferred into (2%) liquid Luria–Bertani (LB) medium containing ampicillin (50 μg ml^−1^) and kanamycin (50 μg ml^−1^), and then incubated at 28 °C in a shaker. When optical density (OD_600_) reached the mid-growth phase (0.5–0.8), cultures were divided into two parts. 0.01% arabinose (final concentration, wt/vol) was added into one part to turn on the cell suicide containment system whereas nothing was added to the other which was used as a control. Both parts continued to be incubated at 28 °C for 15 h, during which samples were taken every 1.5 h. A Beckman DU800 spectrophotometer (Foulerton, MN, USA) and a Hitachi F-4500 fluorescence spectrophotometer (Hitachi, Tokyo, Japan) (excitation wavelength: 488 nm; emission wavelength: 509 nm; bandwidth: 5 nm), were used to detect the OD_600_ and fluorescence of samples respectively.

A plate experiment was performed with the 15-h samples as follows: Samples were diluted with nonselective LB medium till their OD_600_ reached 0.200, then diluted 200 times with the same medium. Finally, one hundred and fifty microliters (150 μl) of the secondary dilution was spread on different selective plates (LB plate containing 50 μg ampicillin ml^−1^, 50 μg kanamycin ml^−1^, or a combination of 50 μg ampicillin ml^−1^ and 50 μg kanamycin ml^−1^). After incubation at 28 °C for 16–24 h, the number of colonies on the plates were counted, and the fluorescence of colonies were detected by using a Leitz DMIRB microscope (Leica, Germany).

### Preparation of cell protein

Cells were harvested by centrifugation at 12,000×*g* for 30 s at 4 °C, washed with 10 mM phosphate-buffered saline (PBS) (pH 7.0), and resuspended completely in the same buffer (200 µl vs. starting volume of 1 ml culture), then the lysozyme of 50 mg/mL and 10% Triton X-100 were respectively added in one percent volume. The suspension was incubated at 30 °C for 15 min, then sonicated on ice until cells break up. The resultant mixture was the total cell protein (TCP), which was then centrifuged at 10,000*g* for 10 min and the supernatant was retained. The supernatant was the crude protein solution (CPS), which was used as the source of the protein to determine the enzymatic activity. Protein concentration was measured using the Bradford method with bovine serum albumin (BSA) as the standard (Bradford 1976).

### Western blotting analysis

Equal amounts of TCP (10 µg) were mixed with SDS-PAGE loading buffer and boiled at 100 °C for 3 min. 10% SDS-polyacrylamide gels was used to separate the protein samples. After electrophoresis, proteins were blotted onto a Hybond enhanced chemiluminescence (ECL) nitrocellulose membrane (Amersham, Arlington Heights, IL, USA) using the wet transblotting method. The primary antibody was anti-GFP monoclonal antibody (Clontech) and the secondary antibody was a horseradish peroxidase-conjugated anti-mouse IgG (Sigma, St Louis, MO, USA). When used, their dilution ratios were 1:1000 and 1:5000 dilution, respectively. The Western blots were detected by using standard ECL reagents (Pierce, Rockford, IL, USA) and imaged by using a ChemiDoc XRS system (Bio-Rad, Hercules, CA, USA).

### Carboxylesterase (CarE) activity assay

Carboxylesterase (CarE) activity in CPS was assayed using β-naphthyl acetate (β-NA) as a substrate (Asperen 1962). About 25 μl of 10 mM β-NA was added into 1, 972.5 μl of 200 mM PBS and 2.5 μl CPS and then incubated at 37 °C for 5 min. The reaction was stopped by adding of 500 μl of stop solution (mixture of 1% Fast Blue B and 5% SDS, 2:5, v/v) and the absorbance was detected at 550 nm. Activities are showed as nmol β-naphthol liberated per min per μg protein.

### Determination of degradation of organophosphorus pesticide

In this study, we chose chlorpyrifos as the tested organophosphorus pesticide since chlorpyrifos has been a widely used pesticide, which is a substitute for highly toxic organophosphorus pesticides that had been banned in China and other countries. Chlorpyrifos has a characteristic absorption peak at 293 nm, and the residue of chlorpyrifos in solution can be determined by ultraviolet spectrophotometry. Five hundred microliters (0.5 mL) of CPS and 0.5 mL of 1 mg/mL chlorpyrifos (dissolved in acetone) were added into the test tube containing 4 mL of 0.05 M Tris–HCl buffer (pH 7.2). After mixing, 2 mL of the mixture was taken out, and 0.5 mL of 50% trichloroacetic acid (TCA) as a stop solution was added, then mixed, and temporarily stored in a refrigerator at 4 °C, which was used as a 0-h sample. After the remaining mixture reacted in a water bath at 37 °C for 5 h, 2 mL of the reaction solution was taken up, and 0.5 mL of 50% TCA was added thereto, which was a 5-h sample. Two milliliters (2 mL) of petroleum ether was separately added to the 0-h and 5-h samples for extraction, and the upper organic phase was taken for measurement. The OD_293_ was measured by a spectrophotometer, and the chlorpyrifos content in the samples were calculated according to the standard curve. The degradation rate of chlorpyrifos was expressed by the percentage of residual chlorpyrifos after 5 h of reaction.

### Determination of degradation of pyrethroid pesticides

The pyrethroid pesticides fenpropathrin, permethrin, and tetramethrin were used to study the degradation activity of pyrethroid pesticides by BL21AI-GBS, and the degradation rate was determined by the residual amount of pesticides after 5- h reaction. The degradation reaction systems were set as follows: 0.5 mL of CPS and 0.5 mL of 1 mg/mL pesticide (dissolved in acetone) were added into the test tube containing 4 mL of 0.05 M Tris–HCl buffer (pH 7.2). After mixing, 2 mL of the mixture was taken out, and 0.5 mL of 50% TCA was added as a reaction terminator, mixed, and temporarily stored in a refrigerator at 4 °C, which was used as a “0-h sample”. After the remaining mixture reacted in a water bath at 37 °C for 5 h, 2 mL of the reaction solution was taken up, and 0.5 mL of 50% TCA was added thereto, which was used as a “5-h sample”. Two milliliters (2 mL) of n-hexane was separately added to the 0-h sample and 5-h sample for extraction, and the upper organic phase was taken for measurement. The pyrethroid pesticides in the extracting solutions were analyzed by an Agilent 6890 gas chromatograph-5976 N mass selective detector (GC–MSD) system (Agilent Technologies Inc., Santa Clara, CA, USA) equipped with a DB-5 capillary column (30 m × 0.25 mm × 0.25 μm, J & W Scientific, CA, USA). The carrier gas was helium at a constant flow rate of 1.2 mL min^−1^. The column temperature was programmed to rise from 100 to 200 °C at 16 °C min^−1^ and then to 300 °C at 8 °C min^−1^ and held for 5 min at 300 °C. One microliter (1.0 μL) of the sample was injected in splitless mode. The injection port, GC-MS interface, and ion source temperatures were maintained at 250, 300, and 200 °C, respectively. The ionization was carried out in the electron impact mode at 70 eV and the data were acquired under selected ion monitoring (SIM) mode.

### Determination of degradation of organochloride pesticide

Plifenate, an organochloride insecticide was used to study the degradation activity of organochloride pesticide by the GEB, and the degradation rate was determined by the residual amount of pesticides after 2-h reaction. The reaction buffer was 0.01 M potassium phosphate buffer (pH 7.5), and the reaction volume was set to 4 mL, including 500 μg of TCP of BL21AI-GBS and 10 μg of plifenate. After mixing, 0.5 mL of the reaction mixture was taken out, which was used as a “0-h sample”. The remaining mixture reacted in a water bath at 30 °C for 2 h, and then 0.5 mL of the reaction solution was taken up, which was used as a “2-h sample”. 0.5 mL of n-hexane was separately added to the 0-h sample and 2-h sample for extraction. Every sample was extracted twice, and the mixture of two extracts (the upper organic phase) was used as the test sample. The amount of plifenate in the extracting solutions was analyzed by an Agilent 7890A gas chromatograph system with electron capture detector (Agilent Technologies Inc., Santa Clara, CA, USA), equipped with a capillary column HP-1701 (60 m × 0.25 mm × 0.25 μm). The carrier gas was high-purity helium. Both the injection port temperature and the detector temperature were 300 °C. The initial column temperature was set at 50 °C for 1 min, and then increase to 190 °C at a rate of 10 °C min^−1^, and then continued to 220 °C at a rate of 2 °C min^−1^ and hold at this temperature for 5 min.

## Results

### Growth curves and change in intensity of fluorescence

**Fig. 2:**
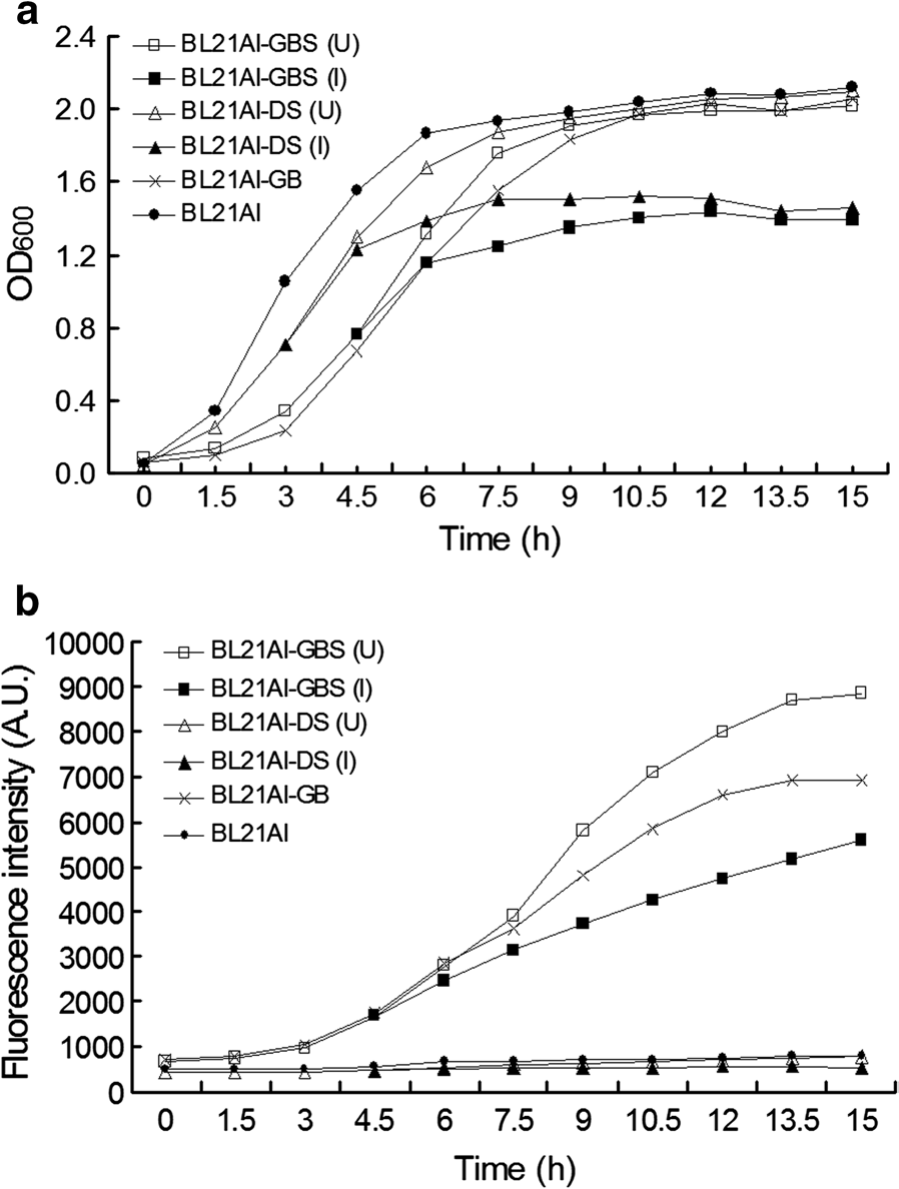
Growth curves (**a**) and fluorescence intensity (**b**) of BL21AI-GBS, BL21AI-DS, BL21AI-GB, and *E. coli* BL21AI^TM^. Cultures of BL21AI-GBS and BL21AI-DS were divided into two parts when their optical density (OD_600_) was in the range of 0.500–0.800. One part was induced by adding 0.01% arabinose, while the other used as a non-induced control. The optical density and intensity of fluorescence of all cultures were detected every 1.5 h with a spectrophotometer and a fluorescence spectrophotometer, respectively. U and I indicate the uninduced culture and induced culture, respectively

Growth curves indicated that cells containing the pDS plasmid (BL21AI-GBS and cells harboring the pDS plasmid alone, hereafter BL21AI-DS) multiplied slightly slower than the controls after the addition of arabinose, and eventually stopping completely about 5 h later of induction (Fig. 2a). To verify the effectiveness of the suicide mechanism, samples collected at 15 h intervals were spread on different selective plates. No colonies of induced BL21AI-GBS cells were found on plates containing kanamycin, or both kanamycin and ampicillin, while 322 ± 18 colonies were found on the plate containing ampicillin. The plates with BL21AI-GBS cultures that had not been induced were densely covered by more than 20,000 colonies (data not shown).

After induction, the intensity of fluorescence of uninduced BL21AI-GBS cultures, and that of cells containing pL-EGFP-B1 (hereafter, BL21AI-GB), increased rapidly with the prolongation of culture time, while the fluorescence of BL21AI-GBS cultures increased more slowly; almost exactly matching its post-induction growth curves (Fig. 2b). Cells without the pL-EGFP-B1 plasmid showed only a basal level of fluorescence during the whole experiment process.

**Fig. 3:**
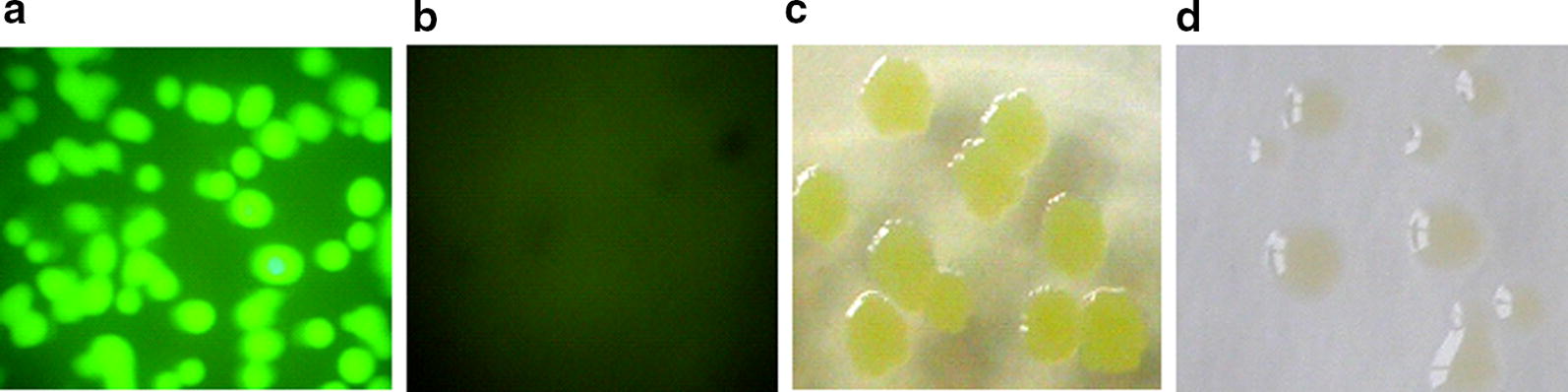
Images of BL21AI-GBS. **a**, **b** Colonies of BL21AI-GBS (**a**) and *E. coli* BL21AI^TM^ (**b**) on LB agar plates photographed by a fluorescent microscope (magnification: 10 × 4); **c**, **d** colonies of BL21AI-GBS (**c**) and *E. coli* BL21AI^TM^ (**d**) on LB agar plates photographed directly in daylight

**Fig. 4:**
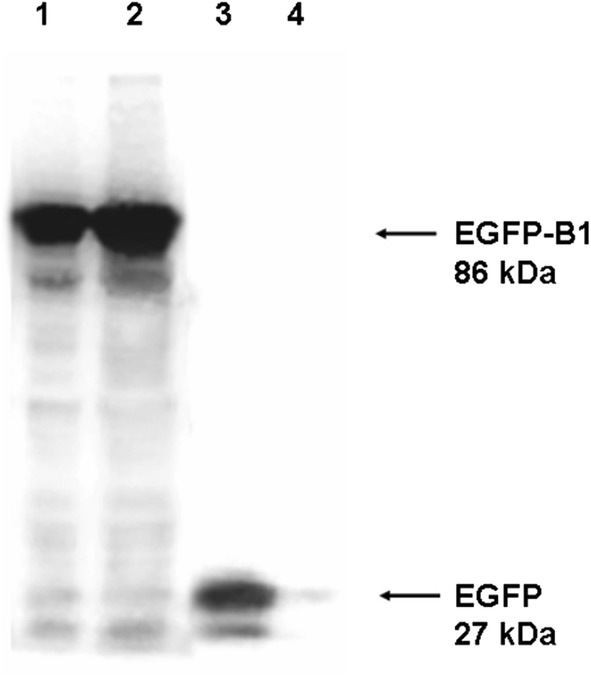
Western blotting analyses of the total cell protein of BL21AI-GBS equal amounts of the total cell protein of BL21AI-GBS, BL21AI-GB or cells harboring pL-EGFP and plasmid-free *E. coli* BL21AI^TM^ were separated by SDS-PAGE and detected with anti-GFP antibody and anti-mouse IgG antibody. Lane 1 to lane 4: samples of BL21AI-GBS, BL21AI-GB or cells harboring pL-EGFP and plasmid-free *E. coli* BL21AI^TM^, respectively

When a fluorescent microscope was used to detect bacterial colonies; strong green fluorescence was detected from colonies of BL21AI-GBS and BL21AI-GB (Fig. 3a, b). Green fluorescence was also detectable in these colonies with the naked eye in daylight (Fig. 3c, d). The Western blot test confirmed the existence of the fusion protein EGFP-B1 in BL21AI-GBS and BL21AI-GB (Fig. 4).

### Carboxylesterase (CarE) activity

β-naphthyl acetate could be used as a substrate to determine the activity of CarE, because the later could degrade β -naphthyl acetate to β-naphthol, which could be estimated by a sensitive colorimetric method. The result showed that CarE activities of the CPS of BL21AI-GBS and BL21AI-GB were 0.634 ± 0.030 and 0.493 ± 0.020 nmol β-naphthol produced per min per μg protein, respectively. However, the basal activity of CarE in *E. coli* BL21AI^TM^ was only 0.082 ± 0.003 nmol β-naphthol produced per min per μg protein (Table 2).

**Table 2:** Carboxylesterase activity of the CPS of BL21AI-GBS, BL21AI-GB, and *E. coli* BL21AI^TM^

Samples	Nanomoles of β-naphthol liberated per min per microgram of protein
BL21AI-GBS	0.634 ± 0.030
BL21AI-GB	0.493 ± 0.020
*E. coli* BL21AI^TM^	0.082 ± 0.003

*CPS* crude protein solution

### Degradation of pesticides

The results of pesticide degradation experiments showed that the residual percentages of the three pyrethroid pesticides (fenpropathrin, permethrin, and tetramethrin) and the organochlorine pesticide plifenate after the degradation by the GEB BL21AI-GBS were significantly different from those of the control bacteria *E. coli* BL21AI^TM^, and there was no difference in the residual percentages of the organophosphorus pesticide chlorpyrifos between BL21AI-GBS and *E. coli* BL21AI^TM^,both were close to 100% (Table 3). The initial and final concentrations of the pesticides used in the degradation experiment were summarized in Additional file 1: Table S1. The 5-h residual percentages of the three pyrethroid pesticides of BL21AI-GBS were below 50% and significantly different from that of the control (*P *< 0.05), while that of the control was all about 100%. Among them, the residual percentages of fenpropathrin and permethrin were less than 20% (18% and 15%, respectively). And the 2-h residual percentage of the GEB for the organochlorine pesticide was about 75%, while that of control was about 95%, and there was also a statistical difference between them (*P *< 0.05) (Table 3; Additional file 1: Table S1).

**Table 3:** Degradation of pesticides by the GEB BL21AI-GBS

Pesticides	Percentage of residual pesticide (%)	
	BL21AI-GBS	*E. coli* BL21AI^TM^
Chlorpyrifos	98.83 ± 7.40	100.48 ± 5.39
Fenpropathrin	17.80 ± 3.00^*^	100.75 ± 18.92
Permethrin	15.04 ± 2.64^*^	106.55 ± 21.73
Tetramethrin	47.00 ± 1.99^*^	108.56 ± 21.37
Plifenate	73.23 ± 7.91^*^	94.58 ± 1.80

^*^P < 0.05, compared with that of control bacteria *E. coli* BL21AI^TM^

## Discussion

As we know, expression of heterologous protein may increase burden on host cells. The gene of fusion protein EGFP-B1 was put downstream of the lambda P_L_ promoter in the recombinant plasmid, and expressed freely in the GEB because of the absence of the lambda *cIts857* repressor. The growth rate of cells containing the plasmid pL-EGFP-B1 (BL21AI-GBS, BL21AI-GB) was slower than others (Fig. 2a), this indicated that the constitutive expression of heterologous protein EGFP-B1 might increase burden on the growth of the GEB. Fortunately, the growth burden did not affect the desired function of the GEB. When inducer was added to trigger the suicide mechanism in the GEB, the growth of BL21AI-GBS and BL21AI-DS decreased significantly and almost stopped about 5 h later of induction.

The results of plate experiment further verified the effectiveness of suicide mechanism. However, the colonies found on plates containing ampicillin but none on plates containing kanamycin indicated that the suicide plasmid pDS was lost in some cells of BL21AI-GBS during incubation, because the resistance of ampicillin and kanamycin was carried by plasmid pL-EGFP-B1 and pDS respectively. To avoid the plasmid-loss, it may be a better way to place the suicide mechanism on the chromosome of host cell or adopt other ways to increase plasmid stability.

The results of fluorescence detection, including detection of fluorescence spectrophotometer, fluorescent microscope and observation by the naked eye in daylight, indicated that the expression of EGFP-B1 endowed BL21AI-GBS with green fluorescence, which could be used to monitor the dispersion and cell density of the GEB (Leff and Leff 1996; Bastos et al. 2001) and indicate the enzyme activity to some extent (Wu et al. 2000).

The results of the enzyme activity assays showed that CPS of the cells containing plasmid pL-EGFP-B1 (BL21AI-GBS, BL21AI-GB) has esterase activity. Notably, the activity of BL21AI-GBS is higher than that of BL21AI-GB. The reason for the difference of activity may be the difference in expression amount or existing form of EGFP-B1 expressed in BL21AI-GBS and BL21AI-GB. Anyway, co-transformation with pDS not only had no adverse effect but also have positive influences on the esterase activity and fluorescence intensity of BL21AI-GBS.

In order to further verify the degradation effect of BL21AI-GBS on pesticides, we chose three types of pesticides organophosphorus pesticide, pyrethroid pesticides and organochloride pesticide to do the pesticide degradation experiments. The results showed that there was no significant difference between BL21AI-GBS and control bacteria in the residual percentage of the organophosphorus pesticide chlorpyrifos after 5-h degradation reaction, but the percentages of residues of the three pyrethroid pesticides fenpropathrin, permethrin and tetramethrin, and the organochloride pesticide plifenate by BL21AI-GBS were significantly lower than that of the control bacteria. And under the same conditions, the residual percentages of permethrin and fenpropathrin were only one-third or less than half of that of tetramethrin, respectively. These results suggested that BL21AI-GBS may have no obvious degradation effect on organophosphorus pesticides under the experimental conditions, but have significant degradation effect on the pyrethroid pesticides and the organochloride pesticide. In addition, the results indicated that the GEB had different degradation effect on different pyrethroid pesticides; the effect on permethrin and fenpropathrin was better than that on tetramethrin. Some researches on GEB of pesticide degradation have been reported, but few of them involve a containment system in the GEB. The co-existence of the containment system and green fluorescence labelling in the GEB generated in our study is more conducive to reduce the environmental risk and improve the possibility of practical application.

In summary, the GEB generated possesses the ability to degrade pesticides, emit green fluorescence and has a containment system. The GEB could be used to degrade various pesticides, easily monitored through green fluorescence when used in practice, and commit suicide when required. Compared with our previous study, the BL21AI-GBS in this study has much broader scope of application, the enzyme activity endowed by EGFP-B1 could be used to degrade various pesticides, including pyrethroid insecticides and organochloride pesticides.

## Supplementary information

**Additional file 1: Table S1.** The concentrations of the pesticides used in the experiment for the bacteria BL21AI-GBS on pesticide degradation.

## Data Availability

All data obtained have been included into the manuscript.
