# Supplementary material for: A safety type of genetically engineered bacterium that degrades chemical pesticides
Source: AMB Express. 2020 Feb 18;10:33. doi: 10.1186/s13568-020-00967-y (PMC7028883; doi:10.1186/s13568-020-00967-y)
Supplement: Supplementary file 1 — Additional file 1: Table S1. The concentrations of the pesticides used in the experiment for the bacteria BL21AI-GBS on pesticide degradation. [file 13568_2020_967_MOESM1_ESM.pdf]

**Suppl. Table S1**. The concentrations of the pesticides used in the experiment for the bacteria BL21AI-GBS on pesticide degradation

| Pesticides | Initial concentrations | Final concentrations |
|---|---|---|
| Chlorpyrifos | 16.22 ± 1.14 (mg/L) | 16.03 ± 2.07 (mg/L) |
| Fenpropathrin | 87.76 ± 12.69 (mg/L) | 15.95 ± 6.72 (mg/L) |
| Permethrin | 77.54 ± 13.91 (mg/L) | 12.01 ± 5.73 (mg/L) |
| Tetramethrin | 93.64 ± 12.90 (mg/L) | 43.98 ± 6.76 (mg/L) |
| Plifenate | 1.63 ± 0.08 (μM) | 1.19 ± 0.15 (μM) |
